# King George's Fund for Sailors

**Published:** 1920-01-10

**Authors:** 


### King George's Fund for Sailors.

DISTRIBUTION OF £50,000.

AT a meeting of the General Council of King George's Fund for Sailors, in the Court Room of the Trinity House, Prince Albert, President of the Fund, presided. He said that it was just over two and a-half years ago, when he was serving at sea, that he received a letter asking him to become the President of the Fund. He thought at the time that the Fund had the prospect of achieving great things for our seamen and their dependants. That prospect was coming to fulfilment. In giving sanction to a further distribution of £50,000 the Fund would have made a total distribution, since its inauguration, of £170,000, a sum which was highly satisfactory.

Captain Clarke said that it was the policy of the Fund to encourage judicious extension among the Marine Benevolent Institutions.

Grants had been approved of £5,000 to the Henry Radcliffe Convalescent Home at Limpsfield, Surrey; £2,500 to the Seamen's Hospital Society towards a Sanatorium for Consumptive Seamen; £2,500 towards the establishment of King George's Merchant Seamen's Hospital at Malta; £2,500 (previously promised) to the Committee of the Marseilles Hospital; £1,000 to the Port Said Hospital for Sailors; and an interim grant of £500 towards the extension of the Alexandra Maternity and Children's Homes. It was now proposed to allot a further sum of £1,000 to the Widows' Pension Fund, administered by the Royal Alfred Institution, and £1,000 to form a Samaritan Fund attached. A further provisional grant of £1,000 was recommended to assist the Grand Fleet Emergency Relief Fund, and a further grant of £200 to the Destitute Fund, administered by the Sailors' Home and Red Ensign Club. The institutions helped are divided into two groups, hospitals and orphanages respectively.

A vote of thanks to his Royal Highness the Prince Albert for presiding at the meeting was proposed by Sir Kenneth Anderson, seconded by Mr. Hartington Ratcliffe, and passed.

